# Expression and distribution of EPHA4 and Ephrin A3 in Aohan fine-wool sheep skin

**DOI:** 10.5194/aab-65-11-2022

**Published:** 2022-01-06

**Authors:** Yu Cui, Chunliang Wang, Lirong Liu, Nan Liu, Jianning He

**Affiliations:** 1 College of Animal Science and Technology, Qingdao Agricultural University, Qingdao, Shandong 266109, China; 2 Nanchang police dog base of the Ministry of public security, Nanchang, Jiangxi 330100, China; 3 China Animal Health and Epidemiology Center, Qingdao, Shandong 266032, China

## Abstract

The objective of this study was to identify the expression and distribution of EPHA4 and Ephrin A3 genes in the development and morphogenesis of hair follicles in fine-wool sheep. The results could lay a theoretical basis for understanding the molecular mechanism that regulates hair follicle development. The skin of Aohan fine-wool sheep at different developmental stages (embryonic day 90, E90d, and 120, E120d, and postnatal day 1, B1d, and 30, B30d) were selected. Real-time quantitative polymerase chain reaction (RT-qPCR) and immunohistochemistry were used to study the levels of mRNA and proteins, respectively. The RT-qPCR results showed that the mRNA expression level of EPHA4 at B1d was significantly lower than at E120d ( p<0.01 ). The expression of Ephrin A3 at E120d was significantly higher than that at E90d and B1d ( p<0.01 ). Immunohistochemical detection results showed that the level and localisation of EPHA4 and Ephrin A3 proteins had spatial and temporal specificity. EPHA4 expression in dermal papilla cells might be important for inducing Aohan fine-hair follicle regeneration and for controlling the properties of the hair. Ephrin A3 might play an important role in the redifferentiation of secondary hair follicles and might also be involved in the inhibition of apoptosis-related gene expression in hair follicles. The Ephrin A3 signalling pathway might accelerate the growth of fine-hair follicles and increase the density of hair follicles.

## 1 Introduction

Wool is a source of high-quality textile raw materials derived from animals that has a significant impact on the national economy (McGregor, 2020). Improving the production of high-quality fine wool has become a hot topic in recent years (Khamiruev et al., 2020). Fine-wool sheep are renowned for their high-quality wool, which is used in the textile industry and is an important agricultural commodity (Guo et al., 2020). Aohan fine-wool sheep is a Chinese sheep breed that produces quality wool and meat (Liu et al., 2013, 2014a, b). Its wool quality is excellent and can be used as a high-grade textile raw material and has a high economic value and representation (Zhao et al., 2020). The organisational structure of the hair follicle and its characteristics have a decisive role in determining the quality and yield of the animal's wool (Driskell et al., 2011; Lv et al., 2020). Hair follicle development is a dynamic process in which the epidermis and dermis exchange signals, and a series of signalling molecules and gene regulation are involved (Lv et al., 2016; Yang et al., 2019; Zhai et al., 2019).

EPHA4 is a member of the erythropoietin-producing hepatocyte (Eph) family, and its ligand is Ephrin A3. EPHA4 receptor is a key component of an extracellular signal transduction pathway and plays a decisive role in the initiation and regulation of biological events (Alonso and Fuchs, 2006; Paus et al., 1999). Crowe et al. (1998) found that EPHA4 is not expressed in label-retaining cells (LRCs) in the carina region of mice. The expression of EPHA4 in the outer root sheath cluster cells of the hair follicles varies greatly through the animal's growth period. EPHA4 is expressed only in the epithelial cells of epidermal hair follicles and spikes. It is then expressed in the newly developed secondary buds and the middle part of the hair follicle (such as the bulge area) during the developmental transition period (day 20) from the resting stage to the growing stage of the hair follicle. In the second growth stage, EPHA4 is downregulated in the hair follicle epithelial cells, upregulated in the hair stromal cells, and not expressed in the hair papilla. Thus, EPHA4 is expressed in skin and growing hair follicle epithelial cells throughout their developmental cycle. EPHA4 is also expressed during feather formation. Its expression precedes the first morphological signal of feather development (Hall, 1998). The expression of EPHA4 is consistent with the changes in the shape of cells in the feather basal plate. Another study showed that the expression of EPHA4 was maintained in the ectoderm. Once the hair bud had a matured polarity, the EPHA4 gene was only expressed in the outer cell layer of the hair buds on the back (Carter, 2010).

Yamada found that the expression of Ephrin A3 is significantly decreased in hair papilla cells of androgenic alopecia patients (Yamada et al., 2008). Ephrin A3 expression is upregulated on day 6 postnatal, peaks at day 14 postnatal, and then decreases abruptly at the end of the growth period. At the beginning of the second growth period, it again increases abruptly. Ephrin A3 is expressed in epithelial cells of the epidermis in the back skin and the hair follicles of the nail of mice that are 0.5 d old. During the transition period (day 20) from the resting stage to the growing stage of hair follicle development, Ephrin A3 is expressed in the newly developed secondary hair bud and the middle of the hair follicle (such as the bulge area). In the second growth stage, Ephrin A3 is highly expressed in the hair stromal cells but not in the hair papilla. The expression of Ephrin A3 increases sharply in the early stage of hair growth, peaks in the middle growth stage, and then decreases suddenly in the resting stage. It was also found that Ephrin A3 protein is expressed in a specific spatiotemporal fashion during hair follicle development. The differentiation process of skin hair follicles in newborn mice injected with Ephrin A3 was significantly accelerated, and the number of hair follicles has significantly increased.

Recent research has revealed that the Eph family in mammalian skin has an important role in the development of follicles and might be involved in the formation of hair follicles (Qazi et al., 2018). Hair follicle development, however, was only studied in humans and mice (Genander et al., 2010). The Eph family expression and the mechanism of function in sheep hair follicle development have not been reported. To understand the role of EPHA4 and Ephrin A3 in the development of fine-wool follicles, we studied the changes in their expression in the hair follicles of Aohan fine-wool sheep. Such knowledge would provide a theoretical basis for the study of the mechanisms that regulate the morphology and development of fine-wool follicles and facilitate accelerated breeding by using molecular biology.

## 2 Materials and methods

### 2.1 Animal care

Aohan fine-wool sheep (AFWS) is a breed of sheep farmed in China for its excellent wool and meat and strong adaptability. The experimental sheep were raised in the AFWS sheep farm of the Inner Mongolia Autonomous Region and fed according to the farm's feeding plan.

### 2.2 Diet and feeding

A total of 12 healthy Aohan fine-wool ewes (aged 3–5 years) were fed under the same conditions, subjected to estrus treatment during September, and artificially fertilised from the same ram. The lateral skin of Aohan fine-wool sheep was collected at gestational days 90 and 120 (E90d and E120d, respectively) and postnatal days 1 and 30 (B1d and B30d, respectively), samples of shoulder skin tissue with (diameter 2 cm) were also collected from the 12 sheep (three samples at each stage). The ewes and lambs were anesthetised with sodium pentobarbital (25 mg kg ${}^{-1}$ ) by intravenous injection. After sample collection, the ewes and newborn lambs were released, whereas the fetuses from E90d and E120d were placed, still under anesthesia, inside a closed chamber and sacrificed by carbon dioxide inhalation. The anesthesia procedure was performed following published protocols. Samples were immediately placed in liquid nitrogen for real-time quantitative polymerase chain reaction (RT-qPCR) analysis.

### 2.3 Test instruments and reagents

TriPure RNA Isolation Reagent, a Transcriptor First Strand cDNA Synthesis Kit, and SYBR Premix EX Taq II (2 × ) were purchased from Roche. The RM2235 slicer and the Feather Microtome Blade 35 slicer were purchased from Leica. The BX51 positive fluorescence microscope was purchased from Olympus. The xylene, anhydrous ethanol, 4 % ethanol polyformaldehyde, 1 % phosphate-buffered saline (PBS) solution, citric acid buffer, and anti-fluorescence attenuation tablets used were all of analytical purity. The primary antibody used was rabbit anti-EPHA4 antibody (BA2275-1). The secondary antibodies used were goat anti-rabbit IgG/Cy3 conjugated antibody, rabbit anti-Ephrin A3 primary antibody (bs-9758R, Bolson), and anti-rabbit IgG secondary antibody (whole molecule)-FITC (Sigma).

### 2.4 Design and synthesis of primers

We used Primer 5.0 software to design EPHA4 (gene ID: 101118852), Ephrin A3 (gene ID: 117506), and *GAPDH* (gene ID: 443005) primers based on the available nucleotide sequences of related sheep genes in GenBank. Biotechnology (Shanghai) Co., Ltd. (Shanghai, China), synthesised the primers listed in Table 1.

### 2.5 Total RNA extraction from skin tissues and quality assessment

Total RNA was extracted from the skin tissues using the Total RNA extraction reagent method, and the quality of the extracted total RNA was assessed using the Bioanalyzer 2100 (Agilent, CA, USA).

### 2.6 Synthesis of complementary DNA (cDNA)

Synthesis of the first DNA strand was done using a reverse transcription kit (Roche) according to the manufacturer's instructions.

### 2.7 Quantitative real-time PCR

The quantitative real-time PCR reaction system included 10 µ L of SYBR Premix EX Taq II (2 × ), 0.5 µ L of each forward and reverse primer (10 µ mol L ${}^{-1}$ ), 1 µ L of DNA template ( > 100 ng), and 8 µ L of sterilised distilled water. Three replicates were run for each sample, and *GAPDH* was used as the internal reference gene. The relative expression of genes was calculated by the 2 ${}^{-\Delta \Delta \mathrm{Ct}}$ method.

### 2.8 Preparation of paraffin section

Fixed skin samples were trimmed into 5 mm × 5 mm × 3 mm pieces and immersed in a PBS buffer solution for 10 h. During immersion, the liquid was changed every hour. The slices were then dehydrated in a series of increasing alcohol concentrations and were then made transparent with xylene. Subsequently, samples were soaked in paraffin I, II, and III for 1 h each. The tissues were then embedded in wax inside a small carton box for subsequent tests. Two sets of sections were prepared. One set was used for immunohistochemical analysis of EPHA4 and Ephrin A3, and the other set was used as blank control.

### 2.9 Fluorescence immunohistochemistry assay

After conventional dewaxing to water, samples were soaked in PBS buffer for 10 min. Following this, 10 µ mol L ${}^{-1}$ of sodium citrate buffer was added to the antigen repair box and the slides were immersed in it. After 15 min at 98 ${}^{\circ }$ C, the glass container with the samples was left to cool naturally to room temperature. We then performed three washes in PBS buffer for 5 min each. After dripping goat serum, the slides were placed in a wet box and sealed in a constant-temperature box at 37 ${}^{\circ }$ C for 20 min. Subsequently, the blocking serum was discarded, and the liquid around the tissue slices was carefully wiped off. The tissue slices were then covered with 1:150 anti-Ephrin A3 and anti-EPHA4 primary antibodies. In parallel, PBS buffer was used to replace the primary antibodies as a negative control. Samples were then washed three times with a PBS buffer for 5 min each, covered with the FITC-labelled goat anti-rabbit IgG antibody, and placed in a wet box at 37 ${}^{\circ }$ C for 30 min. The goat anti-rabbit IgG antibody was discarded, and the slides were washed three times with a PBS buffer for 5 min each. Finally, sections were sealed with an anti-fluorescence attenuator. The slides were observed under a fluorescence microscope, and images were acquired for analysis.

**Table 1:** Primers sequences for EPHA4, Ephrin A3, and *GAPDH* genes.

Gene	Sequence	Size (bp)	Tm ( ${}^{\circ }$ C)
EPHA4	F: AAACTCATCCGCAATCCC	125	60
	R: TGGAGCCAATCGCCTACT		
Ephrin A3	F: GCCCCATCAAGTTCTCGG	137	60
	R: CGGAGTGCGATGTGGAGG		
*GAPDH*	F: AAGTTCAACGGCACAGTCA	125	60
	R: ACCACATACTCAGCACCAGC		

## 3 Results

### 3.1 Total RNA extraction and detection

After extracting total RNA from the skin samples, it was detected by Eppendorf Biophotometer Plus spectrophotometer at an optical density of 260/280 and 1.8–2.0 after running the extracted RNA samples through 1.0 % agarose gel electrophoresis. Both 28S rRNA and 18S rRNA were bright, and the ratio between them was close to 2:1 . The results showed that the total RNA extracted was of good integrity and that the concentration and purity could meet the requirements of subsequent experiments.

### 3.2 Amplification of EPHA4 and Ephrin A3 genes by common PCR

**Figure 1:**
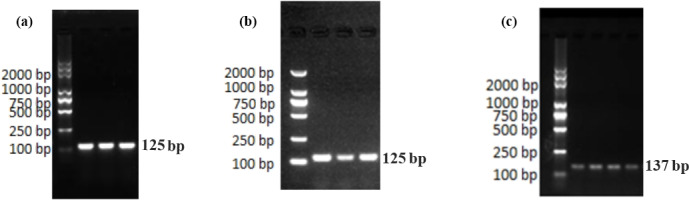
PCR amplification of *GAPDH* **(a)**, EPHA4 **(b)**, and Ephrin A3 **(c)**.

In Fig. 1, we know the results of the PCR amplification of *GAPDH*, EPHA4, and Ephrin A3. The results show that the target bands of primer amplification were well separated, and the size of the PCR products was 125, 125, and 137 bp, which were the same as the target bands.

### 3.3 EPHA4 quantitative real-time PCR amplification

**Figure 2:**
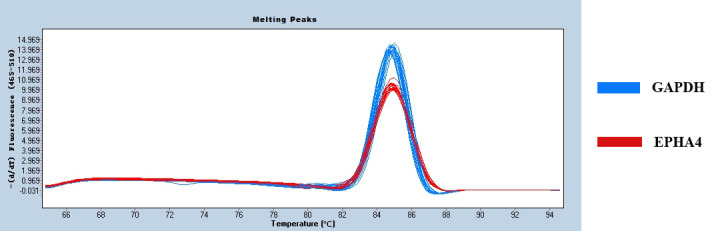
The melting curves of EPHA4 and *GAPDH*.

Figure 2 shows only one obvious peak on the melting curve of EPHA4 and *GAPDH*, which indicates that the fluorescence signals come from specific amplification products. Neither EPHA4 nor *GAPDH* produced non-specific amplification or primer dimers. The data are reliable and can be used for further analysis.

**Figure 3:**
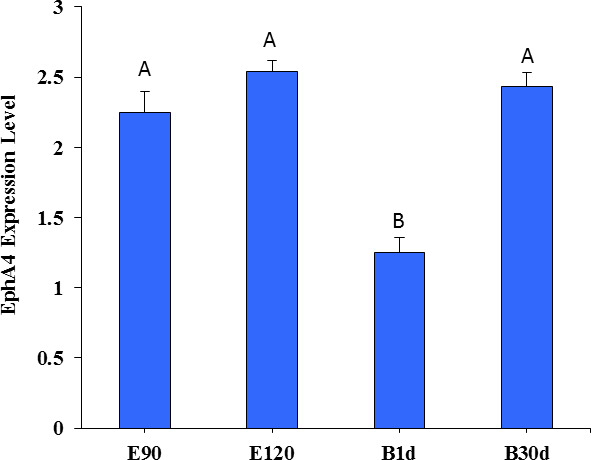
The relative expression level of EPHA4 in skin of fine-wool sheep at different developmental stages.

Figure 3 shows the expression of EPHA4 in fine-wool sheep skin. There was no difference in expression between the E90d, E120d, and B30d groups ( p>0.05 ). The expression was significantly lower in the B1d when compared to that in the other three groups ( p<0.01 ).

### 3.4 Quantitative real-time PCR results of Ephrin A3

**Figure 4:**
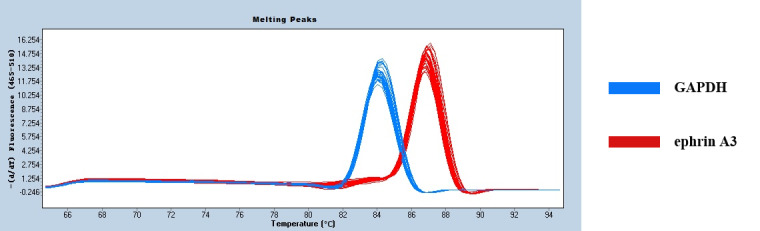
The melting curves of Ephrin A3 and *GAPDH*.

Figure 4 shows only one obvious peak on the melting curve of Ephrin A3 and the internal reference gene *GAPDH*, which indicates that the fluorescence signals come from specific amplification products during the RT-qPCR. Neither Ephrin A3 nor *GAPDH* produced non-specific amplification or primer dimers. The data are reliable and can be used for further analysis.

**Figure 5:**
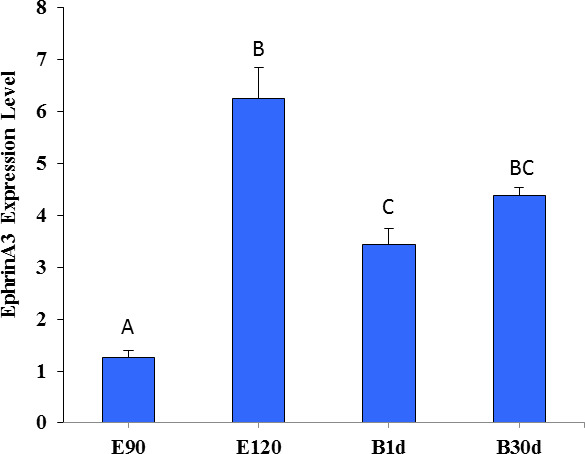
The relative expression level of Ephrin A3 in the skin of fine-wool sheep at the different developmental stages.

Figure 5 shows that the expression of Ephrin A3 in fine-wool sheep skin in the E120d group was significantly higher than in the E90d and B1d groups ( P<0.01 ). There was no difference between the B30d group and either B1d or E120d groups ( P>0.05 ).

### 3.5 EPHA4 protein expression and distribution during skin development of the fine-wool sheep

**Figure 6:**
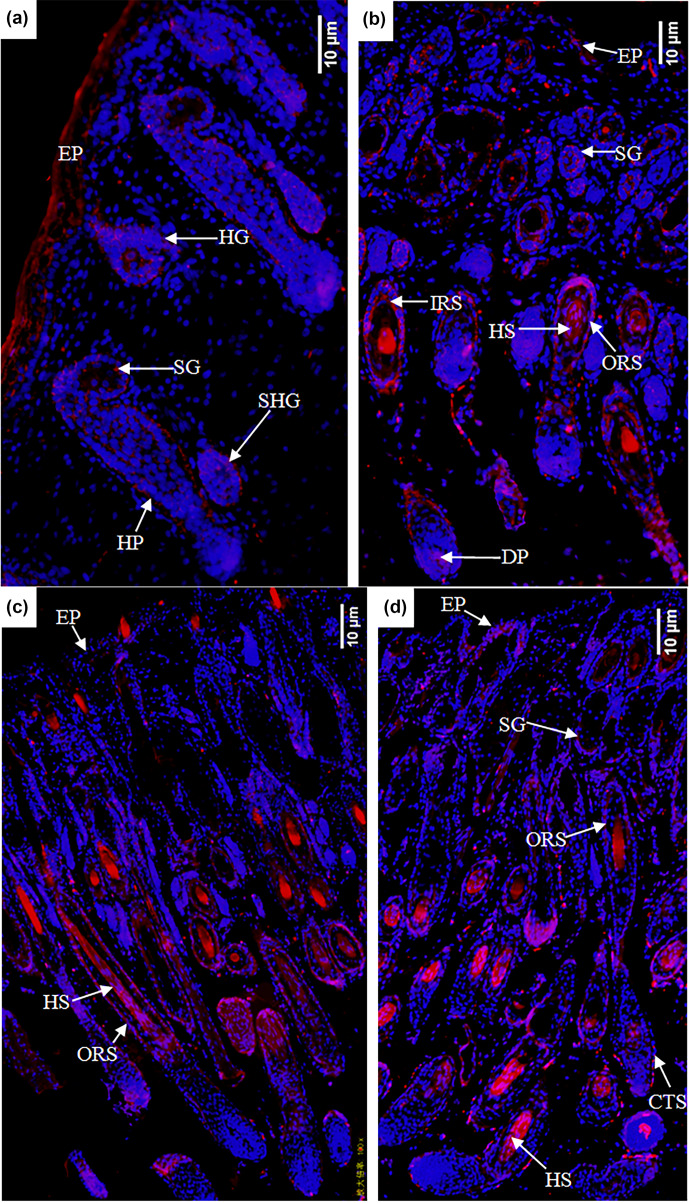
Expression characteristics of the EPHA4 protein in the skin of fine-wool sheep at the different developmental stages: **(a)** E90d group (200 × ), **(b)** E120d group (200 × ), **(c)** B1d group (100 × ), and **(d)** B30d group (100 × ). All samples were collected from the skin on the body side. EP stands for epidermis, HG stands for hair germ, HP stands for Hair peg, SHG stands for secondary follicle germ, HF stands for hair follicle, DP stands for dermal papilla, CTS stands for connective tissue sheath, HS stands for hair shaft, ORS stands for outer root sheath, and SG stands for sebaceous gland.

In the E120d group, EPHA4 protein was found to be expressed in the epidermal cells, primary hair follicle hair buds and hair nail cells, middle and lower cells of secondary hair follicle hair buds, sebaceous gland primordial cells shown in Fig. 6a, hair follicles, outer root sheath cells, hair stem cells, dermal papilla cells, and sebaceous gland cells. There was also little expression in the epidermal cells shown in Fig. 6b. In the B1d group, EPHA4 was mainly expressed in the hair follicle outer root sheath cells and hair stem cells shown in Fig. 6c. In the B30d group, expression was mainly in the hair follicle stem cells, outer root sheath cells, and connective tissue sheath cells. Figure 6d shows that little expression was also noted in the sebaceous gland cells.

### 3.6 Ephrin A3 expression and distribution during skin development of fine-wool sheep

**Figure 7:**
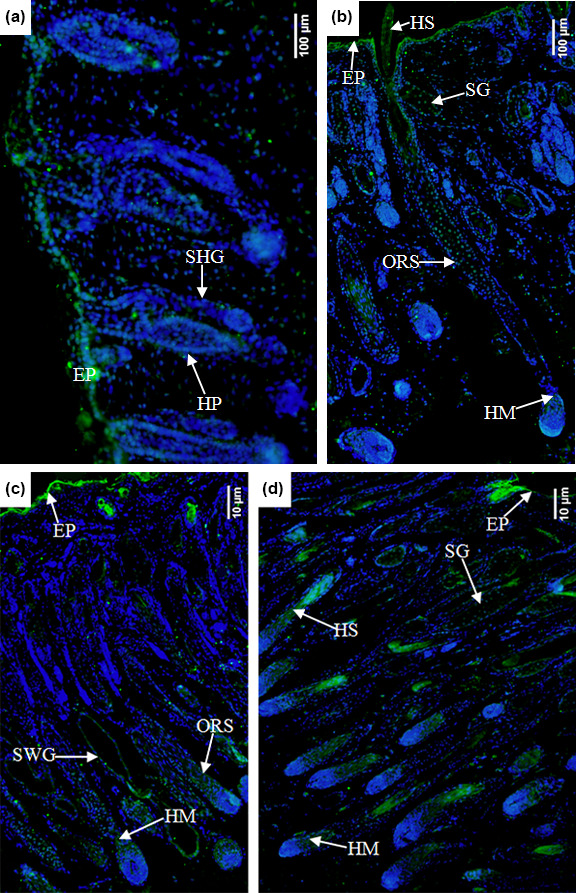
Expression characteristics of Ephrin A3 protein in the skin of fine-wool sheep at the different developmental stages **(a)** E90d group (200 × ), **(b)** E120d group (200 × ), **(c)** B1d group (100 × ), and **(d)** B30d group (100 × ). All samples were collected from the skin on the body side. EP stands for epidermis, HP stands for hair peg, SHG stands for secondary follicle germ, HM stands for hair matrix, SWG stands for sweat gland, HS stands for hair shaft, ORS stands for outer root sheath, and SG stands for sebaceous gland.

In the E90d group, the Ephrin A3 protein was mainly expressed in the epidermal cells and (to a lesser degree) in the hair follicle epithelial cells shown in Fig. 7a. In the E120d group, it was mainly expressed in the epidermal cells, hair stem cells, outer root sheath cluster cells, and hair mother cells. A small amount of the protein was noted in the sebaceous gland cells in Fig. 7b. In the B1d group, it was mainly expressed in the epidermal cells, outer root sheath cells, hair matrix cells, and sweat gland epithelial cells. A small amount was also noted in the epidermal epithelial cells in Fig. 7c. Figure 7d shows that in the B30d group, the protein was mainly expressed in the hair matrix, hair stem cells, and sebaceous gland cells.

## 4 Discussion

The Eph receptors are the largest subfamily in the receptor tyrosine kinase family. It can regulate the functions of the cardiovascular and nervous systems (Ketan et al., 1999). EPHA4, as an important signal transduction protein, plays an important role in cell migration, cell–cell and cell–matrix interactions, cell apoptosis, and proliferation. The EPHA4 receptor is a membrane protein, and only the cell-membrane-bound form is active.

Studies on the Eph receptors indicate that EPHA4 is expressed in the region of feather formation. Moreover, the expression of EPHA4 was reported to precede the first morphological signal of feather development in association with the change of cell shape in the feather basal plate. It was specifically expressed on the feather substrates. Expression of EPHA4 could be restricted by BMP7, and then it would only be detected in normal hair buds. The specific expression of EPHA4 is consistent with its structural specificity. EPHA4 occurs in the basal cells of the ectoderm and is evident in the extension of the epidermis. It was not expressed in labelled cells in specific protuberant areas in mice but was shown to be expressed in the lower outer root sheath cluster cells during the entire growth period. EPHA4 was only expressed in the epithelial cells of the epidermis and hair follicles in mice that are 0.5 d old, while it was expressed in the newly developed secondary hair follicles during the transitional period from the quiescent stage to the growth stage (day 20) of follicular development. During the second growth stage, it is expressed in the middle of the follicle and the downward growing follicular epithelial cells. EPHA4 is highly expressed in hair stromal cells but not in dermal papillae. Therefore, the suggestion that EPHA4 is expressed in epithelial cells of the skin and hair follicles during the entire hair follicle developmental cycle differs from our findings. We show that EPHA4 is expressed in dermal papilla cells by day 120 of gestation. The dermal papilla is composed of dermal papilla cells that are distributed in groups at the base of the hair follicle. It is an induction structure for sending and receiving signals. It controls the size and shape of the hair by controlling the number of hair mother cells. Morphogenesis regulation is the central link, and its main function is to induce hair follicle regeneration. Therefore, it is speculated that EPHA4 might play an important role in inducing the regeneration of Aohan fine-hair follicles and controlling the characteristics of the hair.

Studies have shown that Eph receptors can interact directly with Guanosine triphosphate (GTP) proteins, including ras-GAP regulatory elements. They can interact indirectly with small GTP regulatory elements through Nck (Hall, 1998). In the homeostasis system of the intestinal chorion, notch signalling can induce Ephrin B1 and inhibit EPHB2 expression to establish limits to their action.

The activation of EPHA4 mainly plays a role in cell shape transformation. The expression of follistatin in this region might eliminate the bone morphogenetic protein (BMP)-mediated inhibition on the expression of Eph genes and promote the expression of EPHA4. BMPs also regulate the morphological remodelling of the cells. EPHA4-mediated cell shape changes might be a prerequisite for feather development. In conclusion, local expression of Eph receptors is necessary for the development of local feathers. Our results show that EPHA4 protein is mainly expressed in the outer root sheath cells of the hair follicles at the different developmental stages and expressed in the hair stem cells at all stages, except at day 90 of gestation. It is clear that EPHA4 is indispensable not only in the development of feathers but also in the development of mouse and fine-wool sheep hair follicles. Whether EPHA4 is regulated by BMPs in sheep needs further verification.

In mice, soluble Eph and Ephrin extracellular mimetic peptides could increase the proliferation of hair follicle keratinocytes. Kuroda et al. (2013) found that high levels of dietary vitamin A could enhance Wnt signalling and activate hair follicle stem cells. Overexpression of Wnt in injured skin of adult mice could increase the number of regenerated hair follicles. Activation of the Wnt receptors, i.e. frizzled proteins 1 or 2, could stimulate the expression of Ephrin A3. In this study, quantitative real-time PCR showed that the relative expression of Ephrin A3 was significantly higher at E120d than at E90d. From E90d to E120d, a large number of primary secondary follicles differentiate into secondary follicles. It was speculated that Ephrin A3 might induce the further differentiation of secondary follicles into primary follicles. Secondary hair follicles play an important role, and this process is likely to be regulated by the Wnt signalling pathway. Among the many Wnt signalling pathways, the Wnt/ β -catenin pathway is a classical one. It is in the focus of diverse research activities through which the pathway was shown to play a regulatory role in hair follicle formation, development, and related cell differentiation. The important function of beta-catenin is to promote the formation of hair follicle cells.

Studies have shown that the number of secondary follicles in one-third of fine-wool sheep fetuses increases slowly after the embryonic stage, and sometimes it might also stop. We found that the relative expression of Ephrin A3 mRNA at B1d is significantly lower than that at E120d. Ehama et al. (2013) found that the formation of hair follicles can be induced by the upregulation of Efna3 (Ephrin A3) in cultured hair follicle papilla cells. It can be inferred that Ephrin A3 might be closely associated with the decrease in the proliferation rate of hair follicle cells during this period.

Ephrin A3 expression in dermal papilla cells of androgen-induced alopecia patients is significantly decreased. The expression of Ephrin A3 is consistent with the hair cycle. Ephrin A3 is expressed in the epithelial cells of the epidermis and hair follicles of mice that are 0.5 d old. For comparison, the results of this study show that at E90d Ephrin A3 protein is mainly expressed in the epithelial cells of the epidermis and hair follicles. It is expressed in newly developed secondary hair buds and the middle of the hair follicles during the transitional period from the quiescent stage of hair follicle development to the growing stage (day 20). During the second growth period, Ephrin A3 is highly expressed in the hair stromal cells. The expression of Ephrin A3 increases sharply in the early stage of hair growth, reaching its peak in the middle stage of hair growth, and then it decreases abruptly in the quiescent stage. It was also found that the Ephrin A3 protein is expressed with spatiotemporal specificity during hair follicle formation, development, and morphological transformation. The sex-related expression indicates that Ephrin A3 plays a wide range of functions in the development and morphological alterations of hair follicles, which might be related to their periodicity. Yamada et al. (2008) further showed that the differentiation of hair follicles in the skin of neonatal mice that were injected with Ephrin A3 was significantly accelerated and that the number of hair follicles was significantly increased, suggesting that Ephrin A3 could accelerate the growth and increase the density of hair follicles. Our results show that the expression of Ephrin A3 is consistent with the trend of secondary follicle density alterations, and this density is involved in determining the density of hair follicles. We can deduct from this that the expression of Ephrin A3 is consistent with the trend of hair follicle density. It is speculated that Ephrin A3 might accelerate the growth of fine-hair follicles and increase the density of hair follicles, but further work is needed to verify its function.

Studies have shown that Ephrin A5 can activate the phosphorylation of the EPHB2 receptor, and its activated form can cause nerve conduction (Himanen et al., 2004). Downregulation of EPHA4 expression can reduce the inhibition of intermediate cortical neurons that were induced by Ephrin A3. Ephrin A3 is expressed in the striatum during development and interacts with the EPHA4 receptor during the migration of intermediate cortical neurons. Further studies have shown that Ephrin A3 can prevent migration and differentiation of intermediate neurons through EPHA4. Yamada et al. (2008) found that both Ephrin A3 and EPHA4 proteins were significantly expressed in developing hair follicle epithelial cells and that the expression trend was the same. This is not consistent with the expression trends of Ephrin A3 and EPHA4 observed in the present study. The expression sites of Ephrin A3 and EPHA4 proteins were different. Whether the Ephrin A3–EPHA4 system could be used to control fine-wool development remains to be studied. EPHA4 and Ephrin A3 exhibit temporal and spatial specific expressions during the formation and morphological changes of hair follicles, indicating that they are closely related to the morphogenesis and development of hair follicles and play an important role. EPHA4 is expressed in dermal papilla cells, and it is speculated that it may play an important role in inducing regeneration of Aohan fine-hair wool follicles and controlling hair traits. Ephrin A3 may play an important role in the redifferentiation of secondary hair follicles and may also inhibit the expression of genes related to hair follicle apoptosis. The Ephrin A3 signalling pathway may accelerate the growth rate of fine-wool follicles and increase the density of hair follicles.

## Data Availability

No data sets were used in this article.
